# The effect of Acot2 overexpression or downregulation on the preadipocyte differentiation in Chinese Red Steppe cattle

**DOI:** 10.1080/21623945.2020.1776553

**Published:** 2020-06-24

**Authors:** Lixiang Liu, Jian Wu, Yi Gao, Yang Lv, Jiajia Xue, Lihong Qin, Cheng Xiao, Zhongchang Hu, Lichun Zhang, Xiaotong Luo, Yanli Wang, Yang Cao, Yang Cao, Guoliang Zhang

**Affiliations:** a Branch of Animal Husbandry, Jilin Academy of Agricultural Science, Gongzhuling, China; b College of Animal Science and Technology, Jilin Agricultural University, Changchun, China; c Key Laboratory of Beef Cattle Genetics and Breeding, Ministry of Agriculture and Rural Agriculture, Changchun, China; d Jilin Beef Cattle Breeding and Breeding Technology Innovation Center, Gongzhuling, China; e Jilin Kuncheng Animal Husbandry Technology Development Co., Ltd, Gongzhuling, China

**Keywords:** Acot2, adipocyte differentiation, overexpression, interference

## Abstract

The quality and nutritional value of beef is closely linked to its content of intramuscular fat (IMF). The differentiation of preadipocytes and the deposition of lipid droplets in the adipocytes are the key to regulate the IMF content. The differentiation of adipocytes is regulated by a series of transcription factors and genes. Acyl-CoA thioesterase 2 (Acot2) hydrolyzes the acyl-coenzyme A (CoA) into free fatty acids and CoA and has the potential to maintain the free fatty acids and acyl CoA at the cellular level. In this work, we detected the expression of the *Acot2* gene during the adipocyte differentiation in Chinese Red Steppe cattle, and then interfered and overexpressed the Acot2 gene in the preadipocytes to explore its regulatory role in the adipocyte differentiation. The results showed that the expression and regulation of Acot2 mainly occurred at the later stage of the adipocyte differentiation. The interference with the Acot2 gene significantly inhibited the lipid droplets accumulation and triglyceride content, while its overexpression significantly promoted both of them. The results of this study show that the Acot2 gene is a positive regulator of the adipocyte differentiation and may become a new target to improve the beef quality.

## Introduction

The Chinese Red Steppe cattle is an excellent local breed in Northeast China. Compared with the foreign commercial beef cattle, the content of intramuscular fat (IMF) of the domestic local breeds is lower, and the beef quality and economic benefit are limited [1–3]. The IMF content does not only affect the tenderness and flavour of the beef, but also its nutritional value [4]. The IMF deposition includes a series of processes such as the preadipocyte differentiation, adipocyte maturation, lipid synthesis and decreased muscle growth [5,6]. In the mammalian muscles, triglyceride (TG) is mainly stored in the fat cells in the muscle and the lipid droplets near the mitochondria in the muscle fibres [7,8]. Therefore, the differentiation of preadipocytes and the deposition of lipid droplets in the adipocytes are the key to regulate the IMF content [9].

Previous studies using 3T3 cell lines have shown that the early stages of the adipocyte differentiation are regulated by a series of transcription factors [10,11]. The peroxisome proliferator activated receptor γ (PPARγ) and CCAAT enhancer binding protein α (C/EBPα) are the main transcription factors that regulate the adipocyte differentiation [12–14]. A series of related facts show that the expression of most genes in the process of adipogenic differentiation depends on PPARγ, such as C/EBPα, Srebf1 and Fabp4; thus, PPARγ is considered to be the main adipogenic gene [15,16]. PPARγ and C/EBPα encode the adipocyte specific nuclear hormone receptors, which in turn activate the adipocyte fatty acid binding protein 2 (aP2), lipoprotein lipase (LPL) [17] and carrier proteins that encode the fatty acids, which regulate the expression of adipophenotypic markers and triglyceride accumulation in the middle and late stages of the adipocyte differentiation [18,19].

The acyl-CoA thioesterase 2 (Acot2), also known as MTE-I, PTE2 and ARTISt/p43 [20], is a member of the acyl-CoA thioesterase (Acots) family [21]. It is highly expressed in the mammalian kidney, heart, liver, brain, brown adipose tissue, skeletal muscles and steroid tissues [23,24,25]. In the rodent model of the diet-induced obesity, and compared with the low-fat diet group, the expression of the Acot2 protein in the heart and soleus muscle of the rats that were fed with high-fat diet increased by 2.0 and 7.6 times, respectively [26]. Recently, it has been reported that the interference with Acot2 significantly inhibited the lipid accumulation and triglyceride content in the 3T3-L1 cells [27]. However, the potential regulatory effect of the Acot2 gene on the bovine adipocyte differentiation is not completely revealed. We speculate that the Acot2 gene may play a role in the fat deposition of the Prairie Red cattle. Therefore, we detected the expression of the Acot2 gene during the adipocyte differentiation in the Prairie Red cattle for the first time, and then discussed the regulatory role of the Acot2 gene in the adipocyte differentiation by interfering with and overexpressing it in the preadipocytes. This event may provide a theoretical reference for further studies on the regulatory mechanism of the Acot2 gene on the bovine preadipocyte differentiation.

## Materials and methods

### Experimental animal

The preadipocytes were separated from subcutaneous fat in Chinese Red Steppe cattle and provided by the Animal Husbandry Branch of Jilin Academy of Agricultural Sciences. This research project was examined and approved by the Animal Welfare and Ethics Committee of Jilin Academy of Agricultural Sciences in China (AWEC2017A01, 9 March 2017). The test cattle were handled in accordance with the good animal behaviour that is required by the Animal Ethics procedures and the guidelines of the people’s Republic of China.

### Cell culture and induction of adipocyte differentiation

The cells were resuscitated and inoculated in a 35 mm petri dish (Corning) with a complete culture medium and placed at 37°C in a 5% CO_2_ incubator. The complete culture medium was a Dulbecco’s Modified Eagle’s medium (DMEM) (Gibco) containing 10% foetal bovine serum (Gemini) and 1% penicillin/streptomycin (Sangon Biotech). We changed the complete medium every 2 days. When the cell growth density reached 100% (0d), the complete medium containing 10 mg/mL insulin, 0.5 mM IBMX (Sigma) and 1.0 mM DEX (Sigma) was added to induce the differentiation. The cells were then incubated for 48 hours (2 days) and replaced with a complete medium containing 10 mg/mL insulin, which was changed after 96 hours (4 days). After that, the complete medium was changed every 48 hours until the 8th day of differentiation.

### Oil red O staining

The complete culture medium containing the adipocytes that differentiated to the 8th day was discarded and the adipocytes were washed for three times with PBS buffer and then fixed for 30 min with 4% paraformaldehyde solution (Sangon Biotech) (37°C incubator). We poured out the paraformaldehyde solution, washed it for three times with PBS buffer and then stained the cells with 0.5% oil red O (Sangon Biotech) for 30 min (in a 37°C incubator). We then poured out the oil red O solution and washed it for three times with PBS buffer, then observed the staining with a microscope and took pictures. After taking pictures, the oil red O was extracted with 60% isopropanol, and the optical density was measured at 490 nm.

### Detection of triglyceride content

The triglyceride content of the adipocytes was determined by the triglyceride detection kit (Prilax, Beijing, China) at the room temperature. We poured out the complete medium, washed it for 3 times with PBS buffer, added the appropriate amount of lysate, and sat it at the room temperature for 10 minutes then for another 10 min in a metal bath at 70°C. The supernatant was centrifuged at the speed of 2000 r/min for 5 minutes, and collected for the triglyceride detection.

### Real-time quantitative polymerase chain reaction analysis

Following the manufacturer’s instructions, the total RNA of the cells was extracted using the TRIZOL reagent (Invitrogen), and was then reverse-transcribed into cDNA using a reverse transcription kit (TaKaRa, Japan). Using the prepared cDNA as a template, real-time quantitative polymerase chain reaction (PCR) was performed using the LightCycler® 480II system (Roche). β-actin was used as the internal reference gene, and the specific primers are shown in Table 1. The qPCR reaction system volume is 20 μL: sense and antisense primers 0.5 μL, cDNA 1 μL, SYBR Green IMaster (Roche) 10 μL and H_2_O 8 μL. The reaction procedure is as follows: pre-denaturation at 95°C for 5 min, denaturation at 95°C for 10 s, annealing at 60°C for 15 s, extension at 72°C for 20 s and 40 cycles. The dissolution curve analysis: 95°C for 5 s and 65°C for 1 min. Set 3 repeats for each sample to be tested. The results of the real-time fluorescence quantitative PCR were analysed by the ２^－ΔΔCt^ method.

**Table 1.** Primers for quantitative real-time polymerase chain reaction

Gene	GeneBank	Sequence(5ʹ-3ʹ)	Product size/bp
*Acot2*	NM_001101938.1	F:CTACCTGCTTAATCACCCTC R:GCGGCATAATCTCACCTT	182
*PPARγ*	NM_181024	F:ACCACCGTTGACTTCTCCA R:GGAACCCTGACGCTTTATC	253
*C/EBPα*	NM_176784	F:CGGGAACGCAACAACATCGC R:TGTCCAGTTCACGGCTCAGC	165
*β-actin*	NM_173979	F:GTCCACCTTCCAGCAGAT R:GCTAACAGTCCGCCTAGAA	96

*Acot2*,Acyl-CoA Thioesterase-2; *C/EBPα*, CCAAT/enhancer-binding protein alpha; *PPARγ*, peroxisome proliferator-activated receptor gamma.

### Western blotting analysis

The adipocytes were washed for three times with precooled PBS buffer, and the protein lysate (RIPA) (Thermo Scientific) containing a protease inhibitor (Thermo Scientific) was added. It was cracked on ice for 2 minutes, collected into a 1.5 ml centrifuge tube and cracked again on ice for 30 minutes, during which it was gently shaken for each 5 min. It was then centrifuged at 4°C for 30 min with a rotational speed of 12,000 r/min. Finally, the supernatant was collected, and the protein concentration was quantified using the BCA protein concentration determination kit (Biyuntian, China). The prepared protein samples were denatured at 95°C for 10 minutes, and then the sodium dodecyl sulphate-polyacrylamide gel electrophoresis (SDS-PAGE) was performed. The gel concentration was 10%, and the sample volume of each lane was 20 μg. After the electrophoresis, the protein that was separated from the gel was transferred to a polyvinylidene fluoride membrane (PVDF). At the room temperature, the prepared PVDF membrane was washed on the shaker with tris buffered saline with tween (TBST) containing twitching temperature for 5 minutes, and then sealed with 5% skim milk for 2 hours. After the closure, we washed with TBST for 3 times, 5 minutes each, and then incubated with an antibody on the shaker at 4°C overnight. After the primary antibodies were incubated, the corresponding secondary antibodies (anti-rabbit or anti-mouse IgG-HRP,1:5000) were incubated at the room temperature for 2 hours. The specific information of the primary antibody is shown in Table 2. The chromogenic reaction was carried out using the ECL hypersensitive photoluminescence solution (Pripril, Beijing China), and the protein band information was collected by the ChemiScope 6000 Touch imaging system (Clinx, China). The band strength was estimated by a densitometer and normalized to β-actin (β-actin). The bands were quantified using the Clinx chemical analysis software (Clinx).

**Table 2.** The information of antibodies used in western blot

Gene	Description	Dilution	Source, Number
*Acot2*	Rabbit polyclonal antibody	1:750	Abcam, ab84644
*PPARγ*	Rabbit polyclonal antibody	1:750	Abcam, ab45036
*C/EBPα*	Rabbit polyclonal antibody	1:1000	Sigma-Aldrich, SAB2100396
*β-actin*	Mouse monoclonal antibody	1:5000	Abcam, ab6276

### Acot2 interference sequence design

According to the mRNA sequence of the bovine Acot2 gene (Accession number: NM_001101938.1), which was published by GenBank, we obtained three small interference sequences: Si-810Acot2 (sense: 5ʹ-GCG CUG GCU UAU UAU AAC UTT-3ʹ; anti sense: 5ʹ-AGU UAU AAU AAG CCA GCG CTT-3ʹ), Si-1027Acot2 (sense: 5ʹ-CCU AUG UAG GUG GAA ACU UTT-3ʹ; anti sense: 5ʹ-AAG UUU CCA CCU ACA UAG GTT-3ʹ) and Si-1382Acot2 (sense: 5ʹ-GCA CUA CAU UGA GCC UCC UTT-3ʹ; anti sense: 5ʹ-AGG AGG CUC AAU GUA GUG CTT-3ʹ). The nonsense codon sequence was used as the negative control (Si-NC, sense: 5ʹ-UCC UCU GGA CCA GUG ACC UTT-3ʹ; anti sense: 5ʹ-UCG UGA CUC GUA CCG AGA ATU-3ʹ). Next, we selected one of the best interference sequences for the transfection. All the interference sequences were synthesized by Shanghai Jima Gene Co. Ltd.

### Construction of the Acot2 overexpression vector

The primers were designed according to the protein coding sequence of the bovine Acot2 gene (accession number: NM_001101938.1), which was published by GenBank. Two restriction endonucleases were introduced: HindIII and KpnI, and the complete protein coding sequence of Acot2 was cloned into a PEGFP-N1 vector. The primer was synthesized by Suzhou Jin Weizhi. The PEGFP-N1 vector was used as the negative control (Over-NC).

### Cell transfection

Si-RNA (20 uM) with the best interference efficiency and Si-NC (20 uM) were transfected into preadipocytes. When the degree of the cell fusion reached 75%, the complete medium was replaced with an antibiotic-free medium (petri dish of 35 mm). 5 μl of LIP2000 and Si-RNA (including Si-NC) were respectively diluted with 250 μl serum-free medium. The LIP2000 dilution was placed at the room temperature for 5 minutes, and the Si-RNA (including Si-NC) diluent was placed at the room temperature for 20 minutes, the two were then mixed and added to the antibiotic-free medium. The cells were placed in the incubator (37°C, 5% CO2) and changed to a complete medium that contained 10 mg/mL insulin, 0.5 mM IBMX (Sigma) and 1.0 mM DEX (Sigma) after 12 hours. The cells were incubated for 48 hours (2 days) and replaced with a complete medium containing 10 mg/mL insulin, which was changed after 96 hours (4 days). After that, the complete medium was changed every 48 hours until the 8th day of differentiation.

The Over-Acot2 and Over-NC plasmids were transfected into preadipocytes. When the degree of the cell fusion reached 75%, the complete medium was replaced with an antibiotic-free medium (petri dish of 35 mm). The 2 μg plasmid and 5 μl FuGENE® HD (Promega) were mixed into 100 μl serum-free medium and placed at the room temperature for 15 minutes, the mixture was then dripped into the antibiotic-free medium. The cells were placed in the incubator (37°C, 5% CO2) and changed to a complete medium that contained 10 mg/mL insulin, 0.5 mM IBMX (Sigma) and 1.0 mM DEX (Sigma) after 12 hours. The cells were incubated for 48 hours (2 days) and replaced with a complete medium containing 10 mg/mL insulin, which was changed after 96 hours (4 days). After that, the complete medium was changed every 48 hours until the 8th day of differentiation.

### Data analysis

The results were expressed as mean ± standard deviation. All the experiments were repeated for three independent times. The data processing and picture making were completed using the GraphPad Prism 6.0 software. The greyscale detection of the protein bands was analysed using the Clinx image analysis system (Clinx Science Instruments, China). A value of P < 0.05 was used as the criterion for judging the significance of the difference.

## Result

### Expression of Acot2 and related adipogenic marker genes during the differentiation of bovine preadipocytes

**Figure 1.**
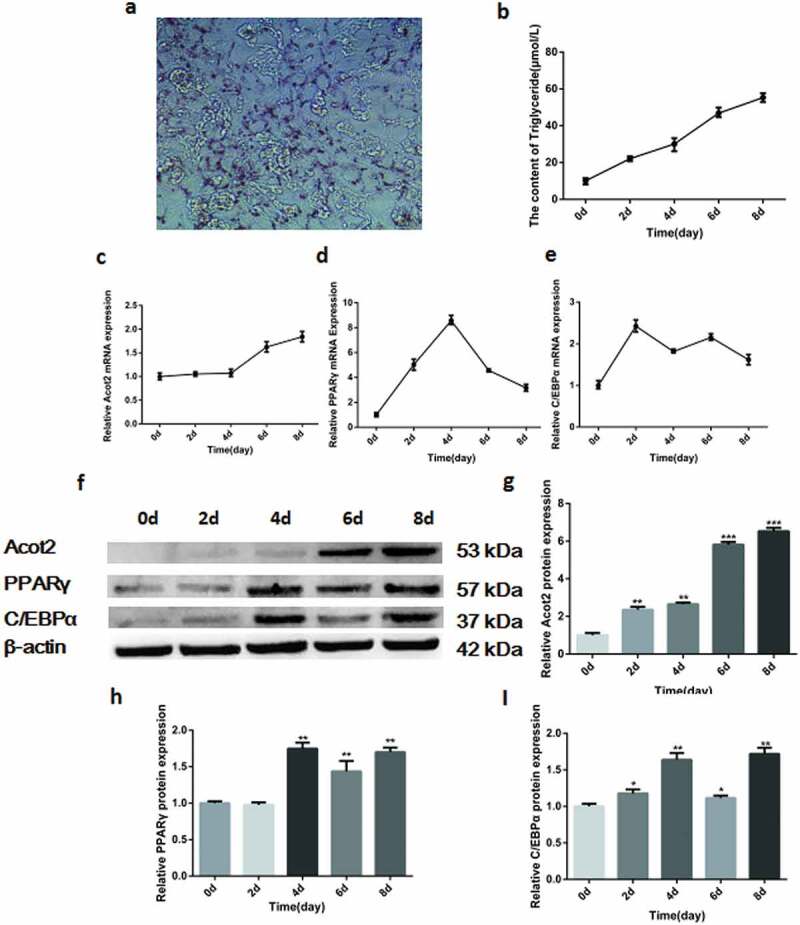
Changes in the mRNA and protein expression of the Acot2 gene and adipogenic marker genes PPARγ and C/EBPα during the adipogenic differentiation. (a) On the 8th day of the adipogenic differentiation with the oil red O staining. The red part represents lipid droplets (200 ×). (b) Changes in the triglycerides during the adipogenic differentiation. (c-e) Changes in the mRNA expression of Acot2, PPARγ and C/EBPα during the adipogenic differentiation. (f) Changes in the protein expression of Acot2, PPARγ and C/EBPα during the adipogenic differentiation. (g-i) The density analysis of the protein immunoblotting (*P < 0.05, **P < 0.01, ***P < 0.001. Acot2, Acyl-CoA Thioesterase-2; C/EBPα, CCAAT/enhancer-binding protein alpha; PPARγ, peroxisome proliferator-activated receptor gamma)

The bovine preadipocytes that were isolated and cultured in vitro showed a large number of lipid droplets after the oil red O staining on the 8th day of differentiation (Figure 1(a)). The triglyceride kit was used to detect the changes in the triglycerides during the adipocyte differentiation, and the triglycerides content gradually increased with the induction of the adipocyte differentiation (Figure 1(b)). The real-time quantitative polymerase chain reaction (PCR) was used to detect the mRNA expression of the target gene during the adipocyte differentiation. The results showed that the expression of the Acot2 gene increased at the late stage of the adipocyte differentiation (Figure 1(c)), while the expression of PPARγ and C/EBPα increased at first and then decreased (Figure 1(d,e)), these results were consistent with the results of the western blotting (Figure 1(f,i)), and they suggest that the bovine preadipocytes were successfully isolated, and that the Acot2 gene may be involved in the late regulation of the adipose differentiation.

### Interference with the Acot2 gene inhibits the preadipocyte differentiation

**Figure 2.**
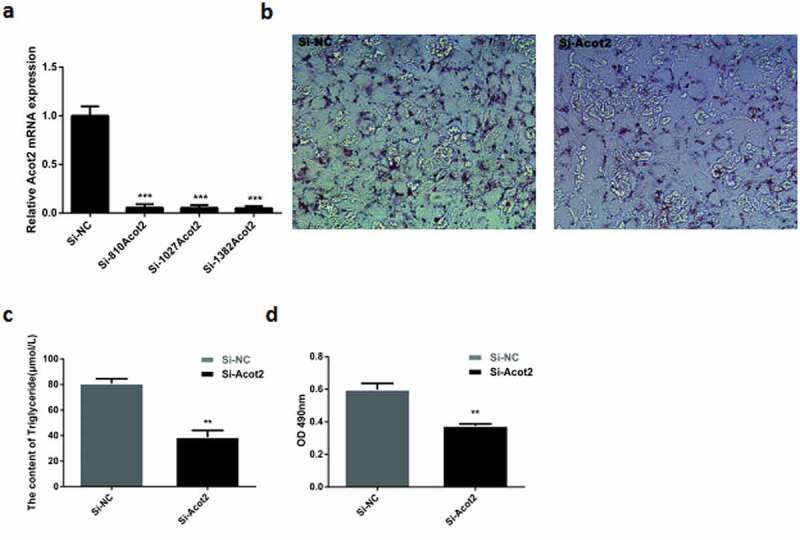
Effects of interfering with the Acot2 gene on the lipid droplet accumulation and triglyceride content. (a) For the interference sequence screening, the Si-1382Acot2 sequence is the best interference sequence. (b) On the 8th day of the adipogenic differentiation with the oil red O staining. The red part represents lipid droplets (200 ×). (c) The content of triglyceride was measured on the 8th day of differentiation in the control and the interference groups. (d) The optical density of the oil red O extract on the 8th day of differentiation was measured in the control and interference groups at 490 nm (lipid droplet quantification) (**P < 0.01, ***P < 0.001, Si-NC is the control group, Si-Acot2 is the interference group)

The sequence Si-1382Acot2 represents the best interference sequence that was selected from the three interference sequences (Figure 2(a)). When the growth density of the preadipocytes reached 75%, Si-NC and Si-1382Acot2 were respectively transfected into the cells. On the 8th day after the adipocyte differentiation, the results of the oil red O staining, triglyceride content and lipid droplet optical density were compared. It was found that the interference with the Acot2 gene significantly inhibited the lipid droplet accumulation and triglyceride content (Figure 2(b-d), P < 0.01).

**Figure 3.**
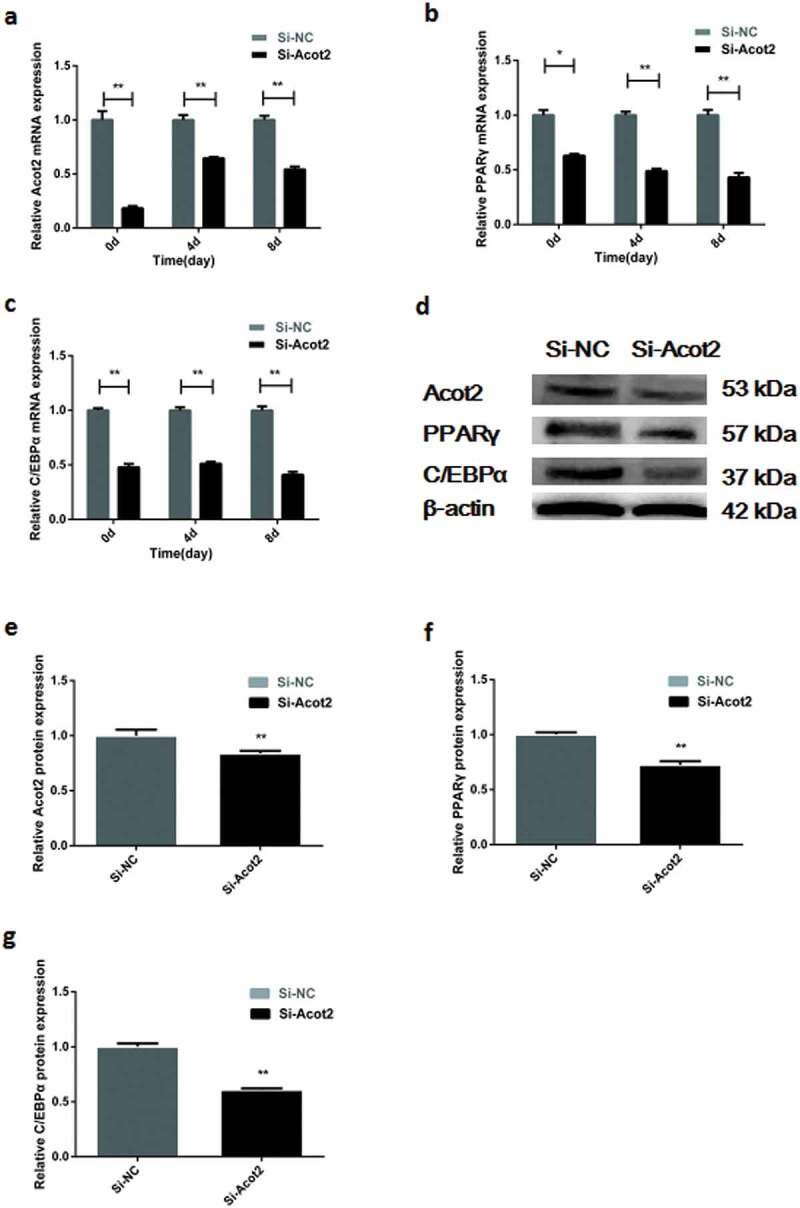
Interfering with the Acot2 gene during the adipogenic differentiation affects the mRNA and protein expression of the PPARγ and C/EBPα genes. (a) Interfering with the change of the Acot2 gene. (b,c) Interfering with the Acot2 gene affects the mRNA expression of PPARγ and C/EBPα. (d) Interfering with the Acot2 gene affects the protein expression of PPARγ and C/EBPα. (e-g) The density analysis of the protein immunoblotting (*P < 0.05, **P < 0.01, Si-NC is the control group, Si-Acot2 is the interference group)

The mRNA expression of the adipogenic marker genes PPARγ and C/EBPα was detected at day 0, 4 and 8 after interfering with the Acot2 gene. The results showed that interfering with the Acot2 gene significantly inhibited the mRNA expression of the PPARγ and C/EBP α genes (Figure 3(a-c), P < 0.01). The protein was detected on the 8th day after interfering with the Acot2 gene. The results showed that interfering with the Acot2 gene significantly inhibited the protein expression of the PPARγ and C/EBPα genes (Figure 3(d-g), P < 0.01).

### Overexpression of the Acot2 gene promotes the preadipocyte differentiation

**Figure 4.**
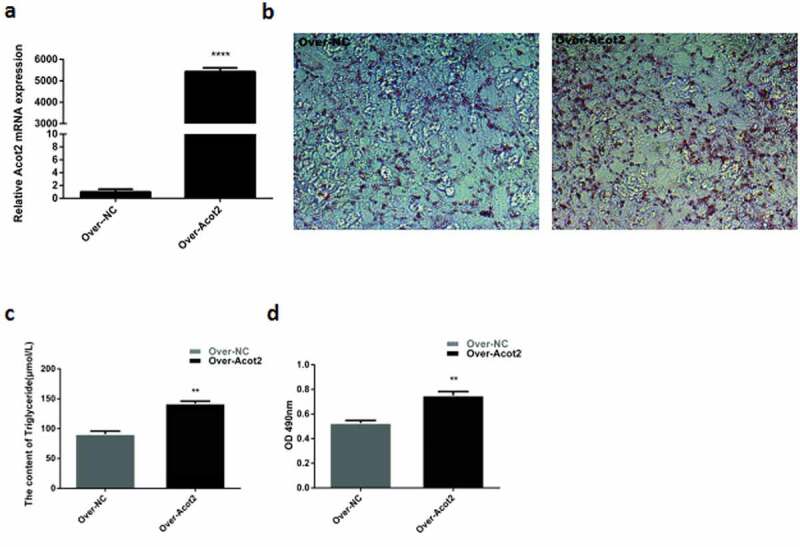
Effects of the overexpression of the Acot2 gene on the lipid droplet accumulation and triglyceride content. (a) Overexpression level verification. (b) On the 8th day of adipogenic differentiation with the oil red O staining. The red part represents lipid droplets (200 ×). (c) The content of triglyceride in the control and overexpression groups was determined on the 8th day of differentiation. (d) Determination of the optical density of the oil red O extract at 490 nm on the 8th day of differentiation in the control and overexpression groups (lipid drop quantification) (**P < 0.01, ***P < 0.001, Over-NC is the control group, Over-Acot2 is the overexpression group)

The detection of the Acot2 overexpression showed that the overexpression efficiency was extremely significant (Figure 4(a), P < 0.001). When the growth density of the preadipocytes reached 75%, Over-NC and Over-Acot2 were respectively transfected into the cells. On the 8th day after the induced differentiation of the adipocytes, the results of the oil red O staining, triglyceride content and lipid droplet optical density showed that the overexpression of the Acot2 gene significantly promoted the lipid droplet accumulation and triglyceride content (Figure 4(b-d), P < 0.01).

**Figure 5.**
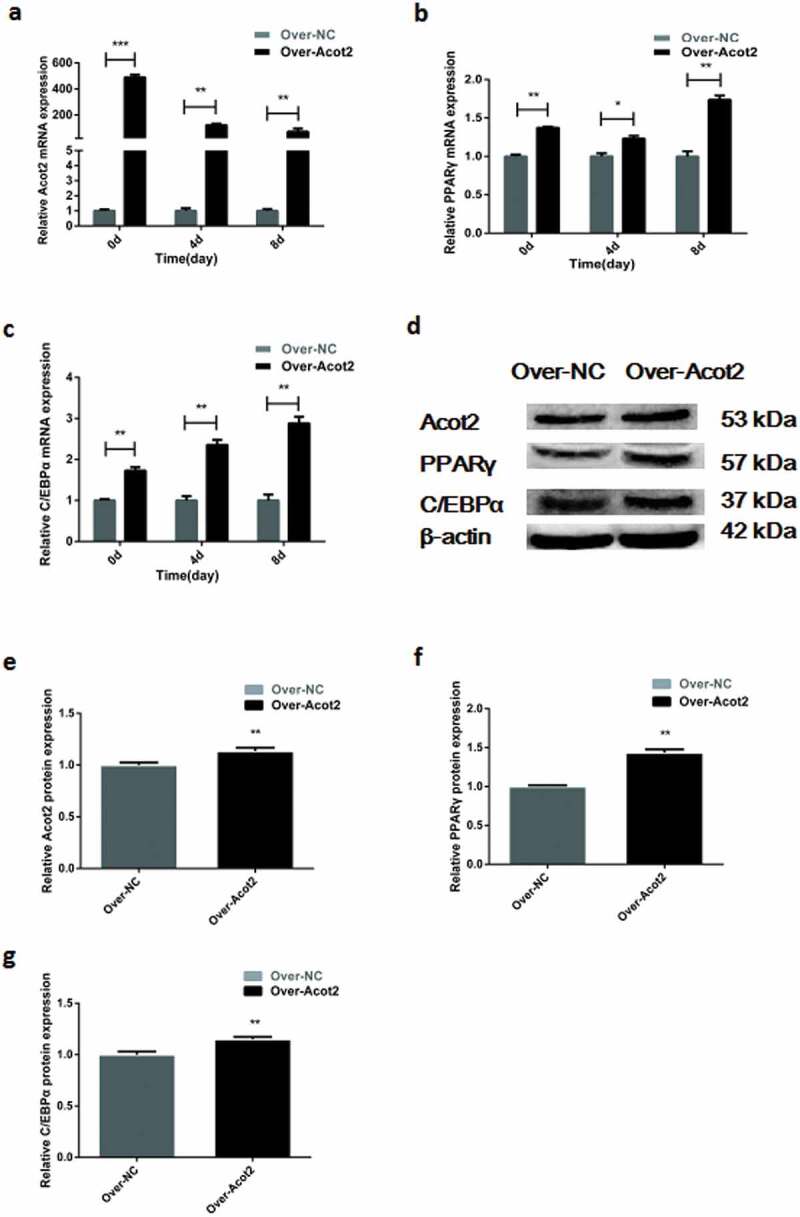
Effects of the overexpression of the Acot2 gene on the mRNA and protein expression of PPARγ and C/EBPα during the adipogenic differentiation. (a) Overexpression with the change of the Acot2 gene. (b,c) Effect of the overexpression of the Acot2 gene on the mRNA expression of PPARγ and C/EBPα. (d) The overexpression of the Acot2 gene affects the protein expression of PPARγ and C/EBPα. (e-g) The density analysis of the protein immunoblotting (*P < 0.05, **P < 0.01, Over-NC is the control group, Over-Acot2 is the overexpression group)

The mRNA expression of the adipogenic marker genes PPARγ and C/EBPα was detected at day 0, 4 and 8 after the overexpression of the Acot2 gene. The results showed that the overexpression of the Acot2 gene significantly promoted the mRNA expression of the PPARγ and C/EBPα genes (Figure 5(a-c), P < 0.01). The protein was detected on the 8th day after the overexpression of the Acot2 gene. The results showed that the overexpression of the Acot2 gene significantly promoted the protein expression of the PPARγ and C/EBP α genes (Figure 5(d-g), P < 0.01).

## Discussion

The Chinese Red Steppe cattle are a long-term cross between the short-horned bull and Mongolian cow. They were accepted by the Chinese Ministry of Agriculture, Animal Husbandry and Fisheries in 1985 and were officially named the Chinese Red Steppe cattle. They belong to the unique breed of milk and meat in Northeast China, and have a good quality, a high content of protein in beef and unsaturated fatty acids, especially the linoleic acid and conjugated linoleic acid, and a delicious flavour that is very suitable for the oriental taste. The quality of the beef is not only affected by the nutritional factors but also restricted by the genetic ones [28]. Therefore, it is of great significance to analyse the genetic factors affecting the beef quality at the molecular level to improve the beef quality and meat quality breeding.

Acyl-CoA thioesterase 2 (Acot2), also known as MTE-I, PTE2 and ARTISt/p43 [20], is a member of the acyl-CoA thioesterase (Acots) family [21], and is highly expressed in the mammalian kidney, heart, liver, brain, brown adipose tissue, skeletal muscles and steroid tissues [23,24,25]. The Acot2 gene does not only promote the oxidation of the mitochondrial fatty acids in the liver [29] but it is also closely related to the adipose differentiation [30]. The research that was done on the Acot2 gene is more reported in mice than in domestic animals and other large mammals. In this study, the expression of the Acot2 gene in the subcutaneous adipocyte differentiation of the Chinese Prairie Red cattle was explored for the first time. The Acot2 gene was mainly expressed and regulated in the later stage of the bovine adipocyte differentiation. The preadipocytes were isolated from the human subcutaneous adipose tissue and induced to differentiate [31]. The mRNA expression of the Acot2 gene was detected and showed that the gene is strongly expressed in the later stage of the adipocyte differentiation [31], while a weak expression of the Acot2 protein was detected in the early stage of differentiation of the rat brown adipocytes. On the 7th day of differentiation, the expression of the Acot2 protein increased to 18 times its expression in the early stage [30]. This may be what the brown adipocytes and white adipocytes have in common. The results of this series of studies show that, as a gene that is involved in the adipocyte differentiation, the expression and regulation of the Acot2 gene mainly occur in the late stage of the adipocyte differentiation.

In order to study the specific effect of the Acot2 gene on the adipocyte differentiation of the Chinese Red Steppe cattle, we interfered with and overexpressed the Acot2 gene in the preadipocytes. The results showed that the interference with the Acot2 gene significantly inhibited the preadipocyte differentiation and lipid droplet accumulation, while these effects were significantly promoted by the overexpression of the Acot2 gene. The studies in the mouse 3T3-L1 cells showed that the interference with the Acot2 gene significantly inhibited the lipid accumulation and triglyceride content in the 3T3-L1 cells [27]. Based on the transcript analysis of the longissimus dorsi muscle and the differentially expressed genes of the intramuscular fat content and fatty acid composition between the Yunling cattle and Chinese Simmental cattle, the expression of the Acot2 gene was found to be upregulated in the Yunling cattle [28]. The expression of the Acot2 gene was also upregulated in the amiodarone-induced mouse model of the drug-induced fatty liver [32]. On the other hand, the overexpression of the Acot2 gene also affected the PPARγ and C/EBPα genes, such that the expression of PPARγ and PPARα was upregulated, and the interference resulted in the opposite effect. The genes of PPARγ and C/EBPα encode the adipocyte specific nuclear hormone receptors, which in turn activate the adipocyte fatty acid binding protein 2 (aP2), lipoprotein lipase (LPL) [17] and carrier proteins that encode the fatty acids, which in turn regulate the expression of the adipophenotypic markers and triglyceride accumulation in the middle and late stages of the adipocyte differentiation [18,19]. These results show that the Acot2 gene plays an important role in the bovine adipocyte differentiation, and it is necessary to further explore the specific mechanism of this role. The results of this study provide a theoretical reference to select the candidate genes that affect the meat quality, thus improve the beef quality.

To sum up, the Acot2 gene is involved in the regulation of the bovine adipocyte differentiation in the later stage and is a positive regulator of the adipocyte differentiation. This result does not only expand our understanding of the regulatory network of the lipid deposition and adipogenic gene expression, but it also suggests that the Acot2 gene may be a new target to improve the beef quality.
